# Selection of Catechin Biosynthesis-Related Genes and Functional Analysis from Chromosome-Level Genome Assembly in *C. sinensis* L. Variety ‘Sangmok’

**DOI:** 10.3390/ijms25073634

**Published:** 2024-03-24

**Authors:** Dong-Jun Lee, Jin-Hyun Kim, Tae-Ho Lee, Myung-Eun Park, Byung-Ohg Ahn, So-Jin Lee, Jeong-Yong Cho, Chang-Kug Kim

**Affiliations:** 1 Genomics Division, National Institute of Agricultural Sciences (NAS), Jeonju 54874, Republic of Korea; leemoses1004@korea.kr (D.-J.L.);; 2 National Agrobiodiversity Center, National Institute of Agricultural Sciences (NAS), Jeonju 54874, Republic of Korea; 3 Research Institute of Climate Change and Agriculture (RICCA), Jeju-si 63240, Republic of Korea; 4 Department of Food Science and Biotechnology, Chonnam National University, Gwangju 61186, Republic of Korea

**Keywords:** chromosome-scale genome, *Camellia sinensis*, catechin biosynthetic pathway

## Abstract

*Camellia* is an important plant genus that includes well-known species such as *C. sinensis*, *C. oleifera*, and *C. japonica*. The *C. sinensis* cultivar ‘Sangmok’, one of Korea’s standard types of tea landraces, is a small evergreen tree or shrub. Genome annotation has shown that Korean tea plants have special and unique benefits and superior components, such as catechin. The genome of *Camellia sinensis* cultivar ‘Sangmok’ was assembled on the chromosome level, with a length of 2678.62 Mbp and GC content of 38.16%. Further, 15 chromosome-scale scaffolds comprising 82.43% of the assembly (BUSCO completeness, 94.3%) were identified. Analysis of 68,151 protein-coding genes showed an average of 5.003 exons per gene. Among 82,481 coding sequences, the majority (99.06%) were annotated by Uniprot/Swiss-Prot. Further analysis revealed that ‘Sangmok’ is closely related to *C. sinensis*, with a divergence time of 60 million years ago. A total of 3336 exclusive gene families in ‘Sangmok’ were revealed by gene ontology analysis to play roles in auxin transport and cellular response mechanisms. By comparing these exclusive genes with 551 similar catechin genes, 17 ‘Sangmok’-specific catechin genes were identified by qRT-PCR, including those involved in phytoalexin biosynthesis and related to cytochrome P450. The ‘Sangmok’ genome exhibited distinctive genes compared to those of related species. This comprehensive genomic investigation enhances our understanding of the genetic architecture of ‘Sangmok’ and its specialized functions. The findings contribute valuable insights into the evolutionary and functional aspects of this plant species.

## 1. Introduction

Tea is one of the most important beverage crops in the world, and it is grown commercially, with more than 6.4 million metric tons of tea produced worldwide in 2021 (https://www.statista.com, 1 October 2022). Regarding the origin of Korean tea, it is believed that Daryum, an envoy of the Silla dynasty, brought tea seeds from China to Samkuksaki in 828 AD. Thus, the history of tea in Korea is a tradition that has been handed down for 1400 years. *Camellia sinensis* cultivar ‘Sangmok’ is a small evergreen tree or shrub that serves as a standard landrace tea in Korea. *Camellia* includes the well-known species *C. sinensis*, *C. oleifera*, and *C. japonica* [1]. Its genome was assembled using the first draft tea genome [2] based on data published in 2017. This Korean tea plant has been found to have particular benefits and superior components, such as catechins, as revealed by genome annotation. Korean tea generally contains numerous secondary metabolites that produce a rich taste and exhibit good efficacy [3]. These metabolites are significantly affected by the gene copy number and expression level [4], particularly key genes essential for the production of three important quality compounds, i.e., catechins, theanine, and caffeine. In this study, we aimed to identify biosynthesis-related genes involved in the catechin biosynthesis pathway, such as those encoding dihydroflavonol reductase (*DFR*), flavanone 3-hydroxylase (*F3H*), leucoanthocyanidin reductase (*LAR*), and anthocyanidin reductase (*ANR*), as well as the serine carboxypeptidase-like (*SCPL*) gene [5], which are associated with the anticancer [6], antioxidant [7], antiaging [7], and antimicrobial [8] activities of the unique Korean tea landrace ‘Sangmok’. Information from this genome sequence can contribute to the understanding of tea genome evolution and metabolomic pathways and facilitate the use of germplasm for tea breeding. The data obtained can be used to identify many candidate genes and functional elements that determine important metabolic pathways related to tea quality and stress resistance, which is important for improving the genetics of cultivated tea plants.

## 2. Results

### 2.1. Chromosome-Level Genome Assembly

**Figure 1:**
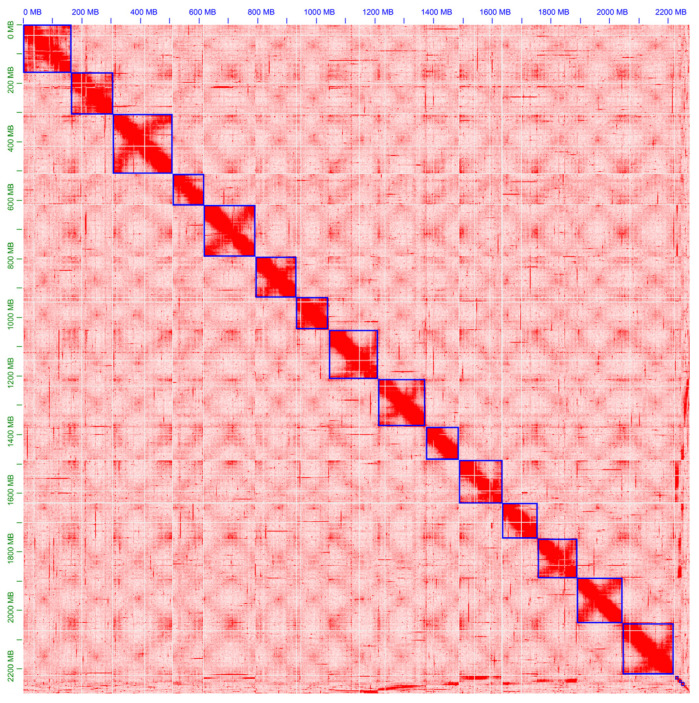
Hi-C contact map of ‘Sangmok’. The raw read pairs were aligned to the genome assembly. Red dots indicate the position of the read pair. Blue boxes with a high density of red dots indicate chromosomes.

**Figure 2:**
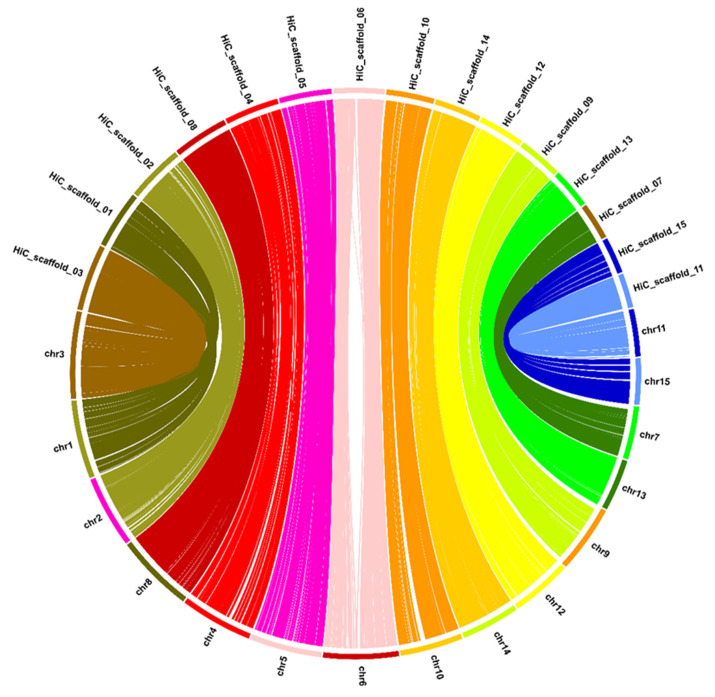
Comparison of the similarity of 15 chromosomes between the NCBI RefSeq genome (chrxx) and the ‘Sangmok’ genome (HiC_scaffold_xx). The lines were connected based on the location of similar sequences and colored differently for each chromosome.

The chromosome-level assembled genome was 2678.62 Mbp long, with a GC content of 38.16%, an N50 scaffold length of 146.06 Mbp, and the longest scaffold length of 201.76 Mbp (Table 1). There were 7171 scaffolds in the ‘Sangmok’ genome assembly, with 15 chromosome-scale scaffolds occupying 82.43% of the assembly (Figure 1, Supplementary Table S1). The ‘Sangmok’ genome was assembled using Pacific Biosciences (PacBio, Menlo Park, CA, USA) long-read technology and Hi-C, as previously reported for the assembly of the *C. sinensis* genome [2,5,9,10]. A total of 15 chromosomes in the ‘Sangmok’ genome were found to align with 15 chromosomes in the NCBI RefSeq genome [10] (Figure 2). We assessed the genome assembly using BUSCO v4.1.2 [11]. Among the 1614 BUSCO groups searched, 1521 and 44 BUSCO core genes were completely and partially identified, respectively, indicating 94.3% BUSCO completeness for the ‘Sangmok’ genome (Table 2). The LTR assembly index was evaluated at 12.91 using LTR_retriever (Supplementary Table S2) [12].

### 2.2. Repetitive Element Analysis

The repetitive element sequences in the ‘Sangmok’ genome were analyzed as tandem repeats and transposable elements (TEs). There are various TE subfamilies in the ‘Sangmok’ genome, including 3.94% DNA transposons, 1.19% LINEs, 0.08% SINEs, 63.26% LTRs, and 12.04% unknown elements, occupying more than half of the genome (Supplementary Table S3). The TE divergence distribution was analyzed using Kimura distances. We found no recent expansion or activation of LTRs (Kimura divergence values ≤ 3; Supplementary Figure S1).

### 2.3. Gene Annotation

**Figure 3:**
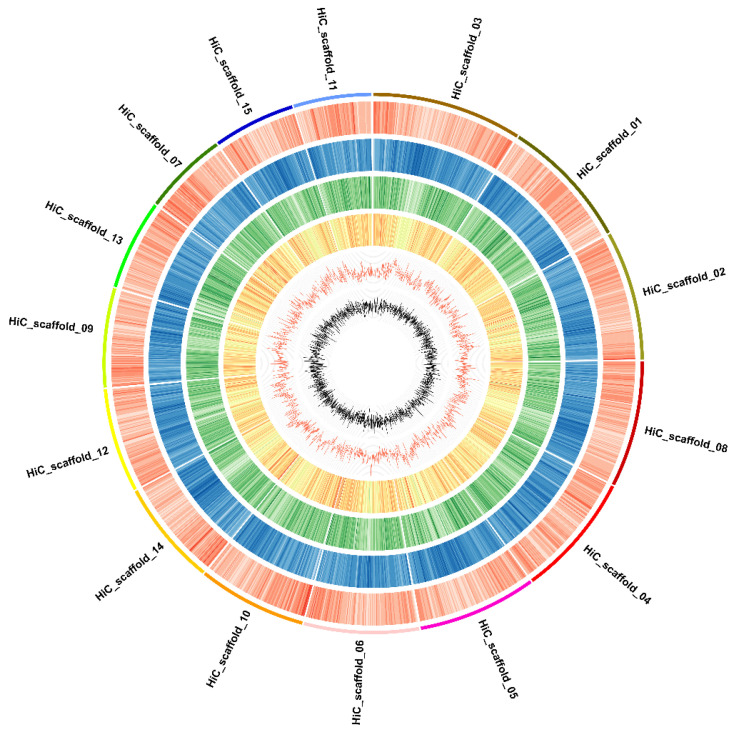
Overview of the ‘Sangmok’ genome. Features are arranged in the order of gene density (black), all-repeat density (brown), LTR/gypsy repeat density (yellow), LTR/copy repeat density (green), GC content (blue), and GC skew (red) from the outside in 1-Mbp intervals across the 15 chromosomes.

We analyzed 68,151 protein-coding genes in the ‘Sangmok’ genome. A total of 85.303 Mbp of coding sequences (CDS) were analyzed, with an average of 5.003 exons per gene. The average exon and intron lengths were 319 and 908 bp, respectively (Figure 3, Table 3). Consequently, 64,033 genes were included in the 15 chromosome scaffolds (Supplementary Table S4). Of the 83,264 CDS, 82,481 (99.06%) were annotated by Uniprot/Swiss-Prot; 71,801 (86.23%) by InterProScan databases [13,14]; and 60,975 (73.23%) by Gene Ontology (GO) functional analysis (Supplementary Figure S2 and Table S5).

### 2.4. Comparison of Gene Families

**Figure 4:**
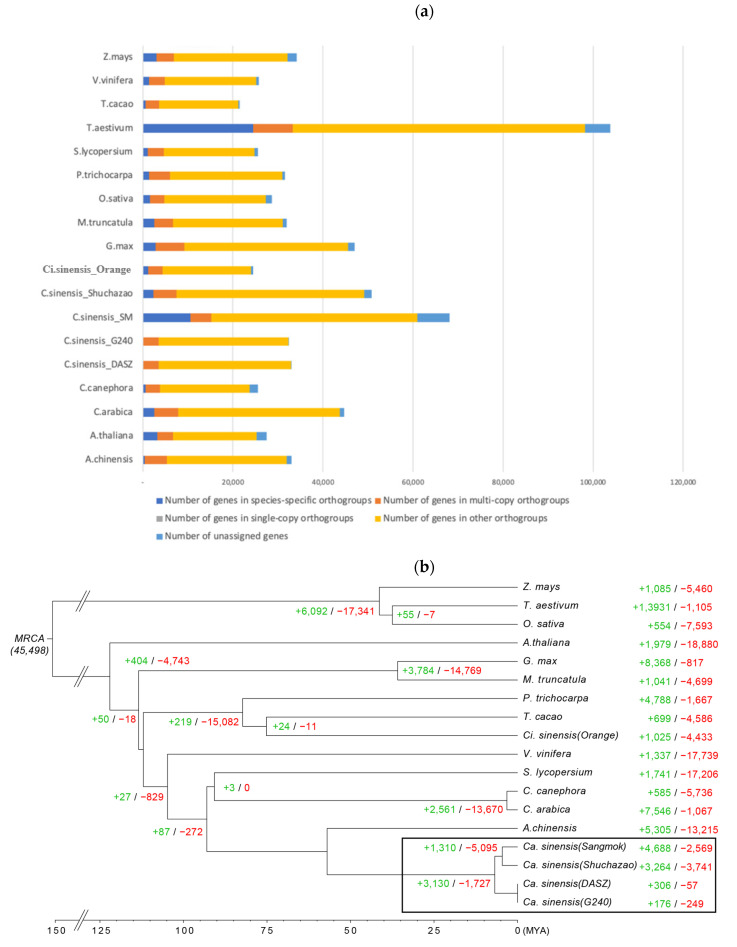
Protein-coding genes in the 18 plant species and phylogenetic analysis. (**a**) Bar graph of the number of protein-coding genes in the 18 plant species, including ‘Sangmok’. The distribution of the number of genes in ‘Sangmok’ compared to the other 17 species by the type of orthogroups is shown. Single-copy orthologs include common orthologs with one copy in all species. Multi-copy orthologs include common orthologs with multiple copy numbers in all species. The number of genes in species-specific orthogroups represents unique genes in specific species. Other orthologs include genes from families shared by 2 to 17 species. (**b**) Phylogenetic analysis of ‘Sangmok’ among 18 plants and gene family gain and loss analysis, including the number of gained gene families (+) and lost gene families (−).

Comparing 18 plant species, including four tea plant species, it was confirmed that the distribution of genes in the orthogroups ranged from 89.5% to 99.9%, and compared to each predicted gene, more than 10% of the genes in the orthogroups of each species were widely distributed in ‘Sangmok’ (15.5%), *Arabidopsis* (11.7%), and wheat (23.6%). The distribution ratio of orthogroup genes in ‘Sangmok’ and species-specific orthogroup genes was higher than that in the other 17 species (Table 4), and 45,498 orthologous gene families from each species were identified among the 18 plant species, with 12,836 gene families being species-specific orthogroups and 60,119 genes being species-specific orthogroups (Figure 4a, Supplementary Tables S6–S8).

### 2.5. Phylogenetic Diversity

A phylogenetic tree of the 18 plants was constructed, where ‘Sangmok’ was most closely clustered with *C. sinensis*, with a divergence time of approximately 160 million years ago (Mya). ‘Sangmok’ and other strains of *C. sinensis* diverged approximately 60 Mya from *Actinidia chinensis*. We found that 4988 gene families were expanded, and 2569 gene families were contracted significantly (Figure 4b). In addition, ‘Sangmok’ and ‘Shuchazao’ diverted from ‘DASZ’ and ‘G240’ at approximately 5 Mya. In ‘Sangmok’, 4988 gene families were expanded, and 2569 gene families were greatly contracted. Each ‘DASZ’ and ‘G240’ were categorized as 306 gene families (+)/57 gene families (−) and 176 gene families (+)/249 gene families (−). In ‘Shuchazao’, which is the closest to ‘Sangmok’, it was found that 3264 gene families were expanded and 3741 gene families were greatly contracted. Moreover, the number of genes in ‘Sangmok’ was high compared to that in the ‘Shuchazao’ species, and the number of genes belonging to the orthogroup was 20% higher on average, indicating the specificity of the ‘Sangmok’ species (Supplementary Figure S3 and Table S9).

### 2.6. Gene Ontology and ‘Sangmok’-Specific Gene Annotation

**Figure 5:**
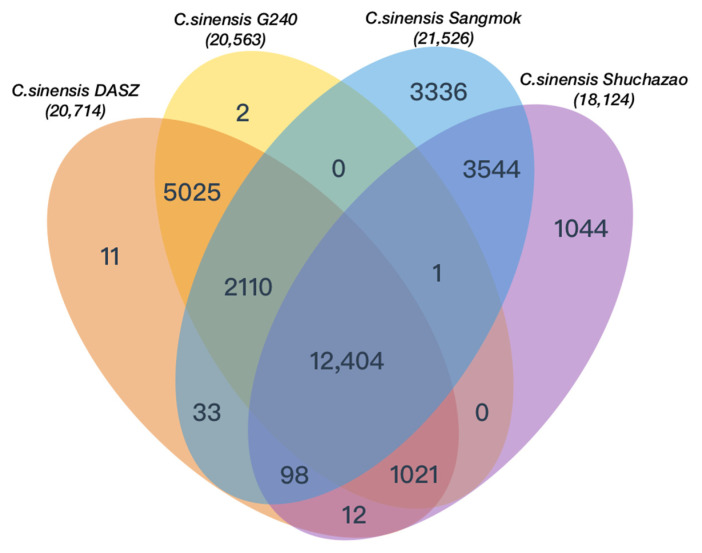
Venn diagram of orthologous gene families between ‘Sangmok’ and three *C. sinensis* strains.

Fisher’s exact test was used to identify GO annotations associated with expanded and contracted gene families (Supplementary Tables S10–S15) and to identify GO annotations for the exclusive families (Supplementary Tables S16–S18). The GO analysis results confirmed that there were many genes involved in auxin transport, multivesicular body assembly, and cellular response via enzymes, such as glucose-6-phosphate isomerase, p-type calcium transporter, and polygalacturonate 4-alpha-galacturonosyltransferase, through the Golgi network. The intersection of orthologous gene families between ‘Sangmok’ and three *C. sinensis* strains was assessed, and 3336 exclusive gene families in ‘Sangmok’ were confirmed (Figure 5). We also extracted 551 similar catechin genes by comparing the protein sequences of previously studied [15] catechin genes with ‘Sangmok’ genomic data and identified 17 ‘Sangmok’-specific genes compared to the exclusive gene families (Supplementary Figure S4 and Table S19). Among the 17 ‘Sangmok’-specific catechin genes, seven genes (*ANR* (1), *F3H* (1), *LAR* (1), and *SCPL* (4)) are involved in catechin biosynthesis; one gene (*DFR*) encodes vestitone reductase (*VR*), which is involved in phytoalexin biosynthesis; and nine genes (*F3H*) are highly related to the cytochrome P450 family, with CYP71 being found in essential oils with monoterpene hydroxylase activity [1], CYP82 being involved in plant defense via seed dispersal and plant pollination by animals [4], CYP85 being associated with a short phenotype [3], and CYP97 exhibiting similar developmental expression pattern among leaves and seedlings [16] (Supplementary Table S20). These genes are critical for elucidating the evolutionary origins and complex biochemistry of ‘Sangmok’-specific genes.

### 2.7. Expression Analysis

**Figure 6:**
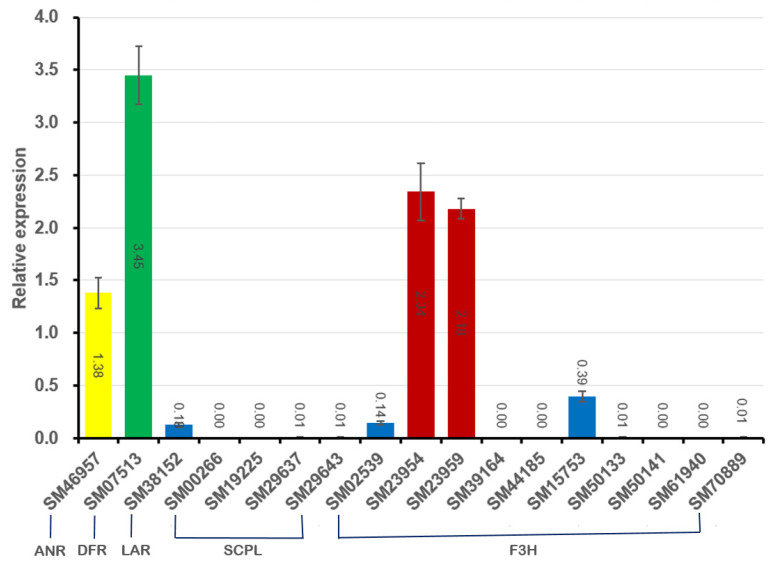
qRT-PCR verification of genes( involved in catechin biosynthesis. The histogram shows the relative expression values (±S.D.) of the genes determined by normalization with CsActin expression obtained through qRT-PCR analyses with three replicates and SM07513 (VR, green), SM46957 (ANR, yellow), SM23954 (CYP97, red), and SM23959 (CYP97, red) were highly expressed and SM38152 (LAR, blue), SM02539 (F3H2, blue), and SM15753 (CYP71, blue) were low expressed.

The expression of the 17 ‘Sangmok’-specific genes reported to regulate catechin biosynthesis (*ANR*, *DFR*, *F3H*, *LAR*, and *SCPL*) was analyzed using qRT-PCR, and the produced histogram shows the relative expression values of the genes normalized to CsActin expression (Supplementary Figure S5 and Table S21). Among these genes, those with relative expression > 1.0, namely, *VR* (SM07513), *ANR* (SM46957), and CYP97 (SM23954, SM23959), were highly expressed, and those with relative expression < 1.0, namely, LAR (SM38152), F3H2 (SM02539), and CYP71 (SM15753), were confirmed to be expressed in small amounts(Supplementary Table S22). *VR* is involved in phytoalexin biosynthesis, plays important roles in plant defense, and is associated with significant health benefits for animals and humans [17]. Moreover, *ANR* is an important gene that is involved in catechin biosynthesis as well as isoflavonoid biosynthesis. CYP97 is involved in leaf and seedling formation and was confirmed to be specifically expressed in ‘Sangmok’ (Figure 6).

## 3. Discussion

The genomic characterization of the Korean tea cultivar ‘Sangmok’ revealed its close evolutionary relationship with other strains of *C. sinensis*, with divergence estimated to have occurred approximately 60 million years ago. Observations of gene family expansions and contractions within the ‘Sangmok’ genome represented pathways implicated in auxin transport and cellular response mechanisms. Furthermore, the discovery of ‘Sangmok’-specific catechin genes with known health-promoting properties, such as anti-inflammatory, antiviral, and antibacterial actions, and defense mechanisms against pests [18] makes ‘Sangmok’ valuable in both agriculture and pharmacology. The remarkable specificity of genes implicated in the catechin biosynthesis pathway within ‘Sangmok’ suggests a unique role in the determination of tea quality and provides fertile ground for comprehensive functional studies. These genes, along with those linked to the cytochrome P450 family, are critical for elucidating the evolutionary origins and complex biochemistry of Korean tea plants. Moreover, they are instrumental in unraveling the sophisticated patterns of leaf and seedling development, which in turn have notable implications for health benefits and antimicrobial activities. For future research, it is imperative to undertake comparative genomic analyses to delineate the distinctive genes of ‘Sangmok’ relative to those present in tea cultivars from other regions. This comparative methodology is likely to shed light on the unique characteristics and lineage of Korean tea cultivars. Additionally, the functional validation of ‘Sangmok’-specific genes and the assessment of their expression patterns under diverse environmental conditions will enhance our understanding of the genomic underpinnings that confer desirable agronomic and medicinal traits in tea plants.

## 4. Materials and Methods

### 4.1. Plant Material

The ‘Sangmok’ cultivar of *C. sinensis*, a standard native Korean tea cultivar, was collected from the Research Institute of Climate Change and Agriculture in Jeju Island, Republic of Korea (33.28 N, 126.31 E), and the PVP Right (Application Number 2011-426) was registered in the Korea Seed and Variety Service (http://seed.go.kr/sites/seed_eng/index.do (19 January 2024), Supplementary Figure S6) for genome sequencing. Young, tender leaves were used for DNA extraction.

### 4.2. Genome Sequencing

We selected both short- and long-read sequencing methods, such as Illumina, PacBio, and Hi-C, for *C. sinensis* genome sequencing.

For short-read sequencing, one paired-end library was constructed using paired-end kits (Illumina, San Diego, CA, USA; Cat#: 20015965) with an insert size of 350 bp. Six mate-pair libraries were prepared using mate-pair kits (Illumina, San Diego, CA, USA; Cat#: FC-132-1001) with average insert sizes of 5, 10, and 15 kb. Sequencing was performed using an Illumina NovaSeq 6000 platform.

For long-read and Hi-C sequencing, we used a Covaris G-tube (Covaris, Woburn, MA, USA) to generate 20 Kb fragments by shearing genomic DNA according to the manufacturer’s recommended protocol. The AMpureXP bead purification system (Beckman Coulter Inc., Brea, CA, USA) was used to remove small fragments. A total of 5 μg of DNA from each sample (DNA quantification was performed using 1% agarose gel electrophoresis and the Qubit dsDNA HS Assay Kit [Thermo Fisher Scientific, Waltham, MA, USA]) was used as the input for library preparation. The SMRTbell library was constructed using the SMRTbell™ Template Prep Kit 1.0 (Pacific Biosciences, Menlo Park, CA, USA; PN 100-259-100). Then, we removed small fragments from the large-insert library using the BluePippin size selection system (Sage Science, Beverly, MA, USA). After the sequencing primer was annealed to the SMRTbell template, DNA polymerase was bound to the complex (Pacific Biosciences, Menlo Park, CA, USA; Sequel Binding Kit 2.0). Purification after polymerase binding was performed using SMRTbell clean-up columns (Pacific Biosciences, Menlo Park, CA, USA; SMRTbell^®^ Clean Up Columns v2 Kit-Mag: PN 01-303-600). The MagBead Kit was used to bind the library complex to the MagBeads before sequencing. MagBead-bound complexes provide more reads per SMRT cell. The polymerase–SMRTbell–adaptor complex was then loaded into zero-mode waveguides. The SMRTbell library was sequenced using 29 SMRT cells (Pacific Biosciences, Menlo Park, CA, USA; Sequel™ SMRT^®^ Cell 1 M v2) and a sequencing kit (Pacific Biosciences, Menlo Park, CA, USA; Sequel Sequencing Kit 2.1), and 1 × 600 min movies were captured for each SMRT cell using the Sequel (PacBio) sequencing platform. The Dovetail Hi-C library was prepared according to the manufacturer’s instructions (Cantata Bio, Scotts Valley, CA, USA; Dovetail Hi-C Library Kit). Young leaf tissues were ground to a fine powder. Plant tissue (250 mg) was crosslinked with PBS/formaldehyde, and chromatin was prepared using SDS and wash buffer. After the plant chromatin sample was normalized, 800 ng of chromatin was used to prepare the library. Chromatin was captured using chromatin capture beads and digested with a restriction enzyme. The end was filled with biotin and ligated to form intra-aggregate DNA. After crosslink reversal, 200 ng of DNA was sheared using a Covaris device. Sheared DNA fragments were end-repaired and ligated with an Illumina adapter. The ligated DNA was purified using streptavidin beads. Purified DNA was amplified by PCR to enrich the fragments. The quality of the amplified libraries was verified using capillary electrophoresis (Bioanalyzer, Agilent, Santa Clara, CA, USA). Sequencing was performed using an Illumina NovaSeq 6000 system following the protocols provided for 2 × 150 bp sequencing.

### 4.3. Transcriptome Sequencing

We prepared 1 μg of pooled total RNA using the SMARTer PCR cDNA Synthesis Kit (Clontech, Mountain View, CA, USA; 634925). Then, the RNA was reverse-transcribed into cDNA. We performed cycle optimization to determine the optimal number of cycles for large-scale PCR using PrimeSTAR GXL DNA Polymerase (Clontech, Mountain View, CA, USA; R050A). After large-scale PCR, AMPure^®^ PB bead purification was performed. For library construction, 1–5 μg of pooled cDNA was used. The SMRTbell library was constructed using the SMRTbell Template Prep Kit 1.0-SPv3 (Pacific Biosciences, Menlo Park, CA, USA; PN 100-991-900). After the sequencing primer was annealed to the SMRTbell template, DNA polymerase was bound to the complex (Pacific Biosciences, Menlo Park, CA, USA; Sequel Binding Kit 2.1: PN 101-429-300). The complex was purified using AMPure purification (Beckman Coulter Inc., Brea, CA, USA) to remove excess primers and polymerase prior to sequencing. The SMRTbell library was sequenced using three SMRT cells per library (Pacific Biosciences, Menlo Park, CA, USA; Sequel™ SMRT^®^ Cell 1 M v2) and a sequencing kit (Pacific Biosciences, Menlo Park, CA, USA; Sequel Sequencing Kit 2.1). The instrument’s operating conditions were 600 min movies with a pre-extension time of 240 min. The Sequel sequencing platform (Pacific Biosciences, Menlo Park, CA, USA) was used to capture each SMRT cell.

### 4.4. Genome Assembly and Hi-C Scaffolding

The FALCON-Unzip assembler [19] was used with the parameters of length cutoff (length_cutoff = 13,000, length_cutoff_pr = 10,000) and filtered subreads from SMRT Link (ver 5.0.0; minimum subread length = 50) for de novo genome assembly. Error correction was performed with short-read data using the Pilon software (ver. 1.23). The draft assembly and Dovetail Hi-C reads were used as input data for HiRise, a software pipeline designed specifically to use proximity ligation data to scaffold genome assemblies. Duplicate regions were removed using Purge_dups [20] to generate a complete chromosome-level scaffold. We constructed a Hi-C library and anchored the scaffolds onto chromosomes after quality control using HiC-Pro [21], Juicer (ver. 1.5) [22], and 3D-DNA (ver. 170123) [23] pipelines based on the draft genome assembly to construct the reference genome at the complete chromosome level. To compare genome sequences, we used MUMmer [24] and Circos [25] with homogeneous coordinates between the two genomes.

### 4.5. Repeat Analysis

A de novo repeat library was constructed using RepeatModeler (ver. 2.0.1) [26], including RECON v1.08 and RepeatScout v1.0.5 [27] software with default parameters. The Tandem Repeats Finder v4.0.9 [28] was used to predict the consensus sequences and classification information for each repeat. All repeats collected by RepeatModeler were used to search the UniProt database [13], where transposon proteins were excluded. To identify highly accurate long terminal repeat retrotransposons (LTR-RTs), we constructed an LTR library using LTR_retriever v2.6 [12] combined with raw LTR data from LTRharvest [29] and LTR_FINDER [30]. Repetitive elements were identified using RepeatMasker with the de novo repeat library, and the repeat landscape was calculated using the Kimura distance for each alignment.

### 4.6. Gene Prediction and Annotation

We performed gene prediction using EVidenceModeler (EVM) v.1.1.1 [31], which integrates the results of multiple gene predictions. The repetitive sequence-masked genome was used for ab initio prediction using Augustus v.3.4.0 [32] and GeneMark-ES v.4.68 [33]. Protein sequences were extracted from the UniProt/Swiss-Prot protein database using ProtHint v.2.6 [33]. These hints were used for protein predictions using GeneMark-EP+ v.4.68 [33] and ab initio predictions using Augustus. The PASA pipeline v.2.3.3 [34] with IsoSeq data was used to obtain transcriptome-level evidence. We used full-length transcriptome data based on the PacBio long-read technology, which produces RNA-based evidence that greatly contributes to the accuracy of gene prediction [35,36]. EVM was used to integrate the ab initio, transcriptome, and protein prediction results to obtain a final gene prediction. The predicted genes were annotated by aligning them with the NCBI non-redundant protein (nr) database [37] and Uniprot/Swiss-Prot using NCBI BLAST v.2.9.0 [38] with a maximum e-value of 1 × 10^−5^. To obtain protein domain information, InterProScan v.5.44.79 [14] was used, with a protein sequence translated from a transcript. GO [39] terms were assigned to the genes using the BLAST2GO pipeline [40] with NCBI BLAST results.

### 4.7. Gene Family and Phylogenetic Analysis

To construct the phylogenetic tree, we first extracted the longest isoform protein sequences from the gene prediction information using custom scripts for each species. A phylogenetic tree of the 17 plant species was constructed using Orthofinder v2.5.4 [41] with an e-value cutoff of 1 × 10^−5^ and an all-to-all BLASTP analysis of 17 plant species. Gene family clustering was performed using the OrthoMCL software v22-282 [42]. We performed multiple-sequence alignment of each gene family using MAFFT [43] and inferred the phylogenetic tree using FastTree [44]. The divergence time was estimated using PATHd8 [45] based on calibration with published divergent times of nodes estimated from fossil evidence or the TimeTree website [46]. The expansions and contractions of gene families were identified by comparing the statistics of gene counts for each gene family between the ancestor and 18 plant species using CAFE software v.4.2.1 [47] with parameters including a *p*-value threshold of 0.01 and automatic searching for the λ value. We selected ‘Sangmok’-specific gene families from the clustering results of four *C. sinensis* species containing ‘Sangmok’ and extracted protein sequences. We performed BLASTP with known catechin protein sequences to select genes with identity > 90% and query coverage > 80%.

### 4.8. qRT-PCR Analysis

qRT-PCR analysis of gene expression was performed as described previously [48]. Total RNA from the tissues of tea plants was isolated using an RNA isolation kit (APureTM Plant RNA kit, APBIO, Seoul, Republic of Korea) according to the manufacturer’s instructions. Approximately 4 µg of total RNA was digested with RNase-free DNase I (Promega, Madison, WI, USA), and the RNA concentration was determined by a NanoDrop ND-2000 UV spectrophotometer (Thermo Fisher Scientific). The first-strand cDNA was synthesized from 2 µg of total RNA with the iScript cDNA synthesis kit (Bio-Rad, Hercules, CA, USA). All cDNA samples were diluted 5-fold for qRT-PCR reactions. Gene-specific primers are listed in Supplementary Table S22. The tea plant CsActin (housekeeping gene) was used as an internal control for the genes determined by normalization with CsActin expression. qRT-PCR data were generated using a CFX Opus Real-Time PCR System (Bio-Rad) in 40 cycles (95 °C for 20 s and 60 °C for 30 s). To identify PCR products generated in the presence of SYBR Green, a Tm analysis was performed by increasing the temperature from 40 to 95 °C at a linear transition rate of 0.1 °C/s.

## 5. Conclusions

In this study, we constructed a chromosome-level genome assembly of *C. sinensis* (L.) Kuntze var. *sinensis* cultivar ‘Sangmok’, a Korean tea landrace. Genomic evaluation was performed using BUSCO (94.3% BUSCO completeness). The final total genome size was 2679.62 Mbp, and a super-scaffold was assembled for 15 pseudo-chromosomes.

We classified orthologous gene families in ‘Sangmok’ and three *C. sinensis* species and confirmed 3336 exclusive gene families in ‘Sangmok’. By comparing these exclusive genes with 551 similar catechin genes, 17 ‘Sangmok’-specific catechin genes (including ANR, DFR, F3H, LAR, and SPCL) were identified. Of these, nine genes related to cytochrome P450 (CYP71A, CYP82C, and CYP85A) derived from F3H were identified as being involved in pest defense mechanisms and in anti-inflammatory, antiviral, and antibacterial mechanisms.

## Figures and Tables

**Table 1:** Summary of the ‘Sangmok’ genome assembly.

	Final Assembly
Number of scaffolds	7171
Total bases of scaffolds (bp)	2,679,620,961
Number of scaffolds > 1 M nt	38
Number of scaffolds > 10 M nt	15
N50 scaffold length (bp)	146,057,547
L50 scaffold count	8
GC content (%)	38

**Table 2:** Assessment of the ‘Sangmok’ genome assembly.

	BUSCOs	BUSCOs (%)
Complete BUSCOs	1521	94.3
Complete and single-copy BUSCOs	1310	81.2
Complete and duplicate BUSCOs	211	13.1
Fragmented BUSCOs	44	2.7
Missing BUSCOs	49	3

**Table 3:** Summary of gene predictions for the ‘Sangmok’ genome.

	Number of Features	Total Length (bp)	Avg. Length (bp)	Density (Mbp)
Gene	68,151	236,339,063	3467.87	25.4331
CDS	83,264	85,302,684	1024.48	31.0731
Exon	416,542	133,000,454	319.297	155.448
Intron	333,278	302,686,548	908.21	124.375

Average number of exons per gene: 5.003; CDS: coding sequence.

**Table 4:** Orthogroup genes and species-specific orthogroup genes in 18 species.

Species	Genes	Orthogroup Genes (%)	Unassigned Genes (%)	Species-Specific Orthogroup Genes (%)
*C. sinensis* (Sangmok)	68,151	60,983 (89.5)	7156 (10.5)	10,561 (15.5)
*C. sinensis* (DASZ)	33,021	32,945 (99.8)	76 (0.2)	38 (0.1)
*C. sinensis (G240)*	32,356	32,328 (99.9)	28 (0.1)	11 (0.0)
*C. sinensis* (Shuchazao)	50,822	49,150 (96.7)	1672 (3.3)	2382 (4.7)
*A. chinensis*	33,044	31,933 (96.6)	1111 (3.4)	404 (1.2)
*A. thaliana*	27,543	25,319 (91.9)	2224 (8.1)	3236 (11.7)
*C. arabica*	44,747	43,739 (97.7)	1008 (2.3)	2583 (5.8)
*C. canephora*	25,574	23,753 (92.9)	1821 (7.1)	635 (2.5)
*Ci. sinensis* (Orange)	24,525	24,045 (98.0)	480 (2.0)	1241 (5.1)
*G. max*	47,013	45,609 (97.0)	1404 (3.0)	2832 (6.0)
*M. truncatula*	31,917	31,077 (97.4)	840 (2.6)	2555 (8.0)
*O. sativa*	28,708	27,272 (95.0)	1436 (5.0)	1564 (5.4)
*P. trichocarpa*	31,618	30,966 (97.9)	652 (2.1)	1401 (4.4)
*S. lycopersium*	25,557	24,836 (97.2)	721 (2.8)	1087 (4.3)
*T. aestivum*	103,785	98,250 (94.7)	5535 (5.3)	24,544 (23.6)
*T. cacao*	21,507	21,174 (98.5)	333 (1.5)	627 (2.9)
*V. vinifera*	25,787	25,166 (97.6)	621 (2.4)	1362 (5.3)
*Z. mays*	34,221	32,196 (94.1)	2025 (5.9)	3056 (8.9)

## Data Availability

The ‘Sangmok’ (*Camellia sinensis* L.) genome sequencing data and genome assembly data are available in NCBI under BioProject: PRJNA939251.

## Supplementary Materials

The following supporting information can be downloaded at https://www.mdpi.com/article/10.3390/ijms25073634/s1.
