# Supplementary material for: Selection of Catechin Biosynthesis-Related Genes and Functional Analysis from Chromosome-Level Genome Assembly in C. sinensis L. Variety ‘Sangmok’
Source: Int J Mol Sci. 2024 Mar 24;25(7):3634. doi: 10.3390/ijms25073634 (PMC11011610; doi:10.3390/ijms25073634)
Supplement: Supplementary file 1 [file ijms-25-03634-s001.zip › Supplementary_Figures(S1-S6).pptx]

## Slide 1
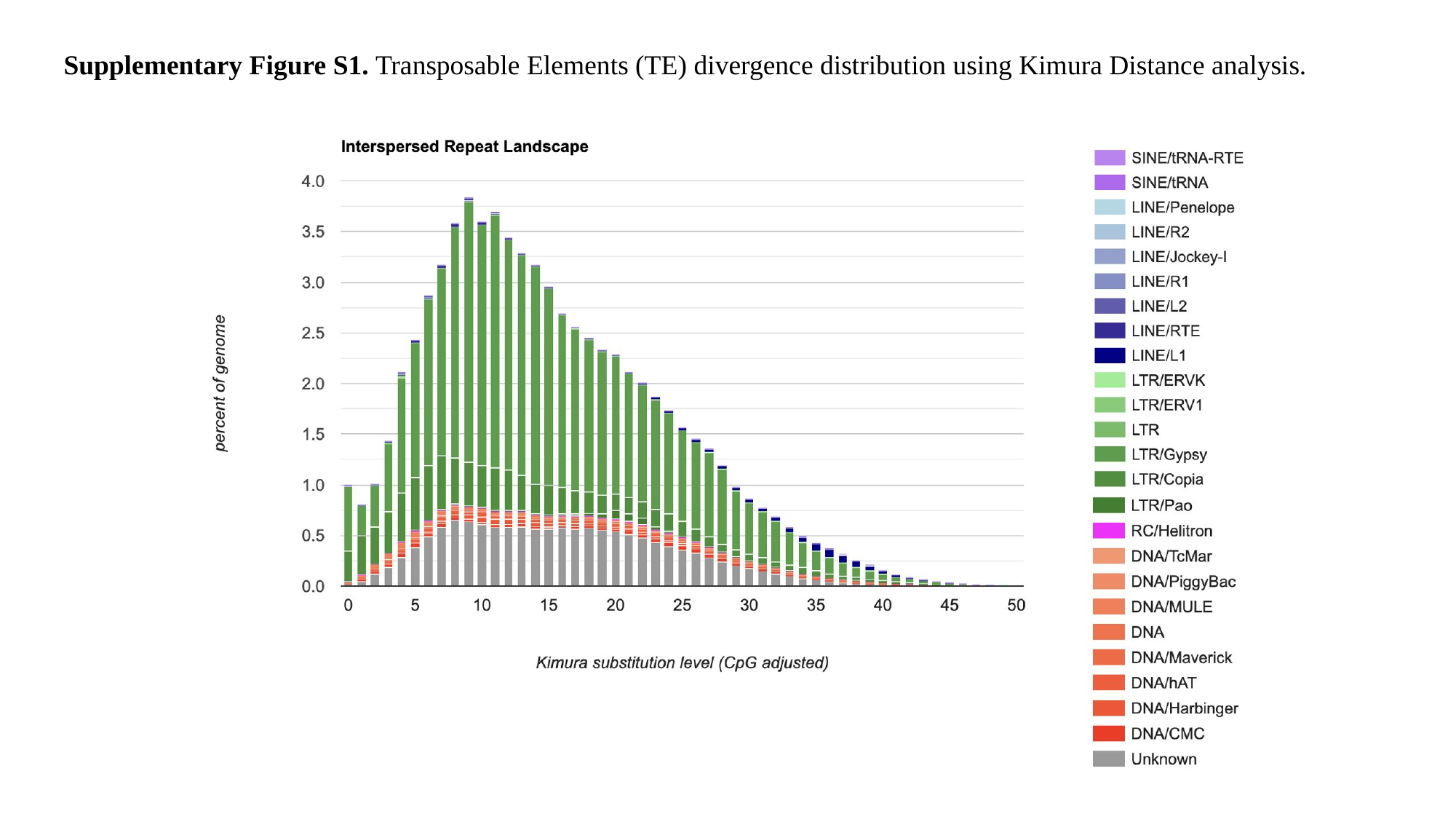

Supplementary Figure S1. Transposable Elements (TE) divergence distribution using Kimura Distance analysis.

## Slide 2
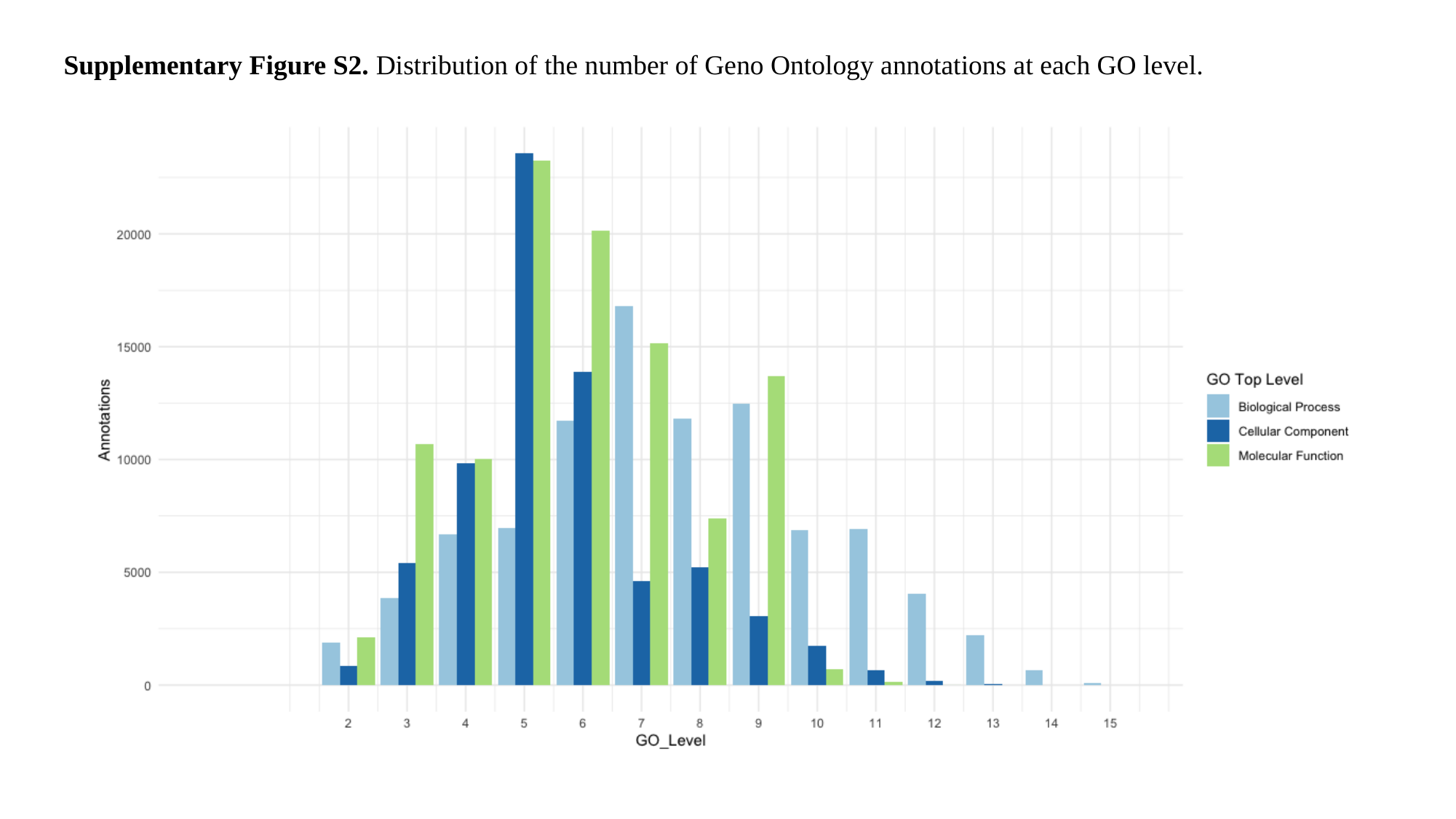

Supplementary Figure S2. Distribution of the number of Geno Ontology annotations at each GO level.

## Slide 3
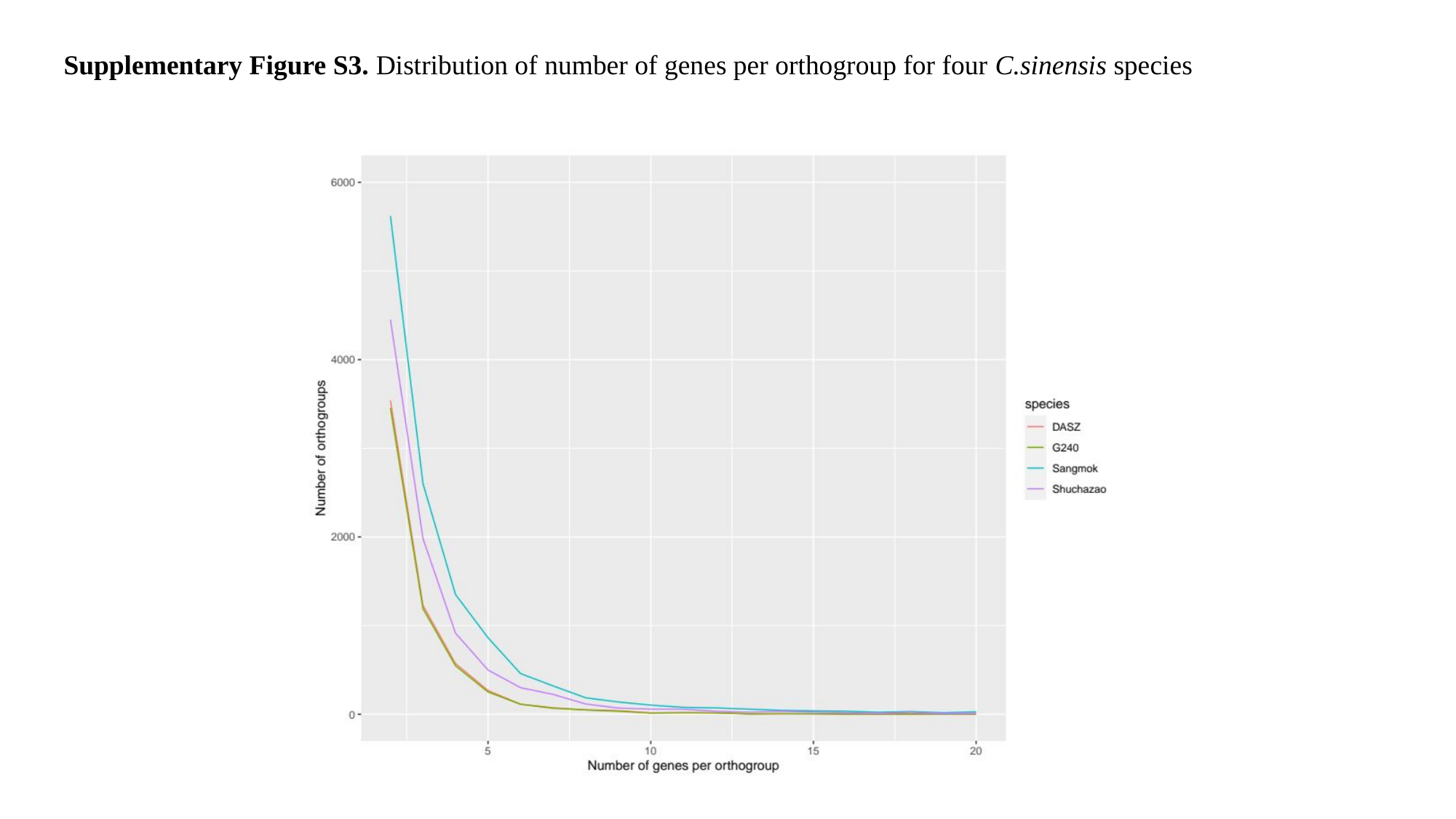

Supplementary Figure S3. Distribution of number of genes per orthogroup for four C.sinensis species

## Slide 4
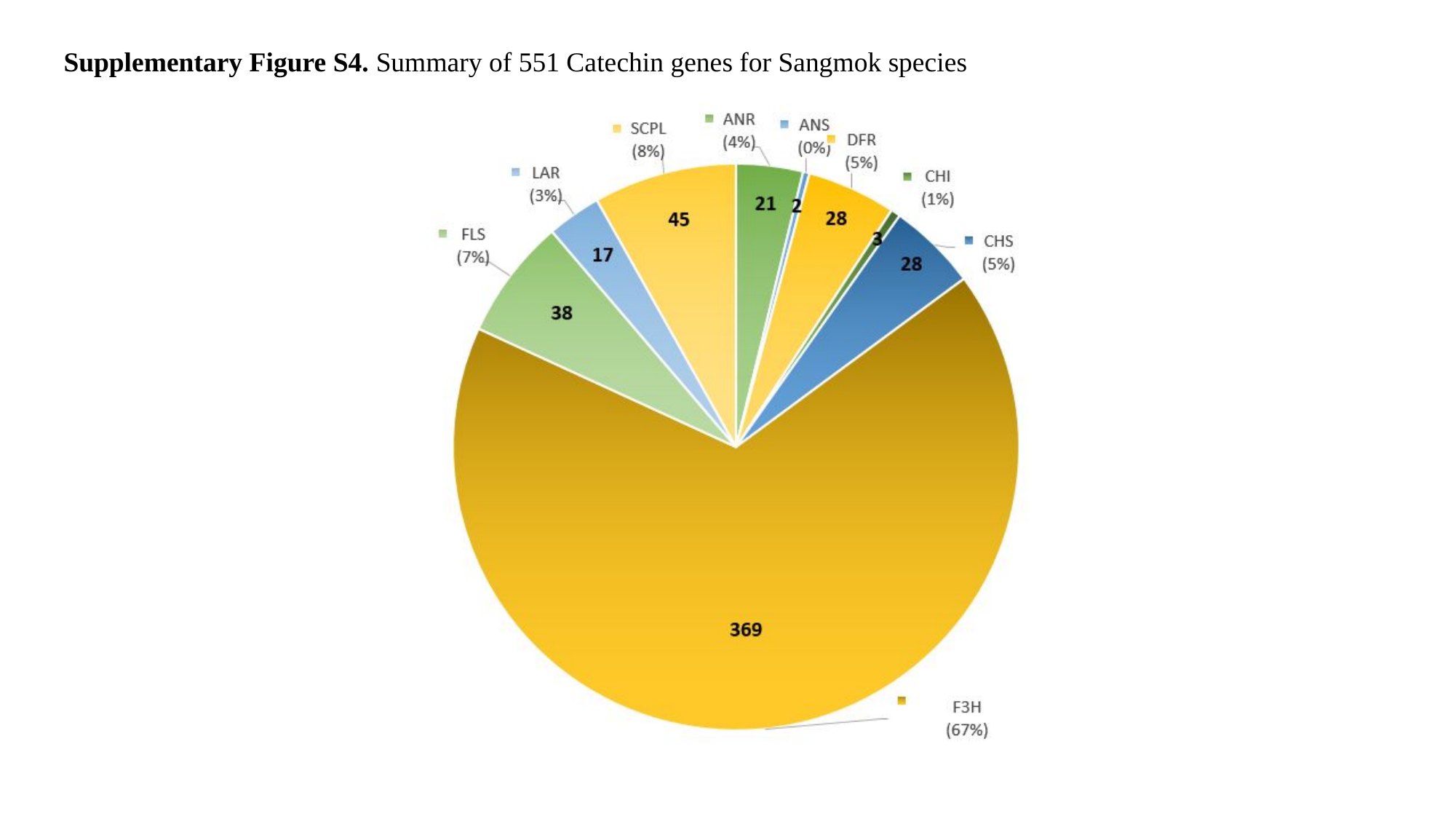

Supplementary Figure S4. Summary of 551 Catechin genes for Sangmok species

## Slide 5
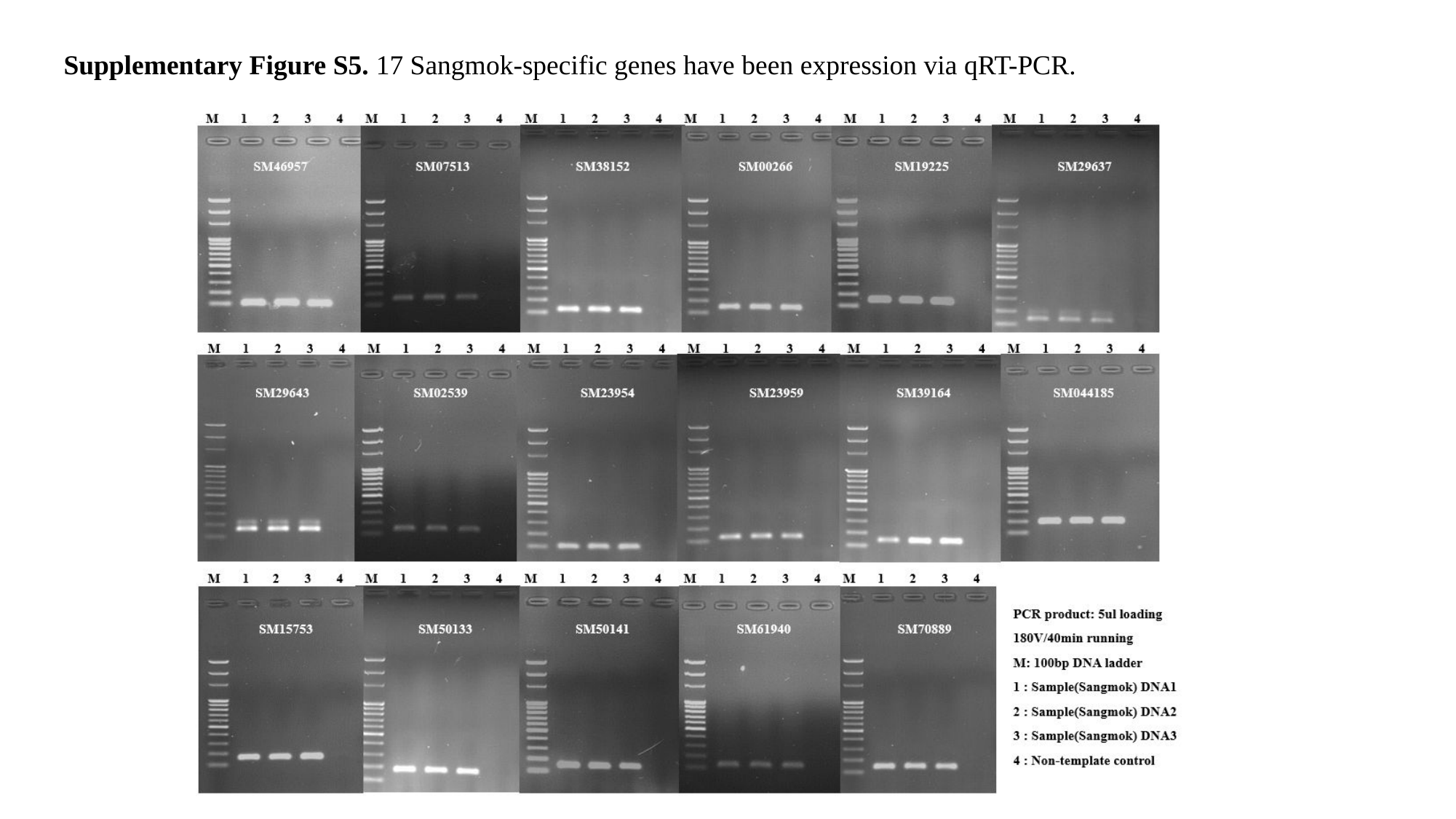

Supplementary Figure S5. 17 Sangmok-specific genes have been expression via qRT-PCR.

## Slide 6
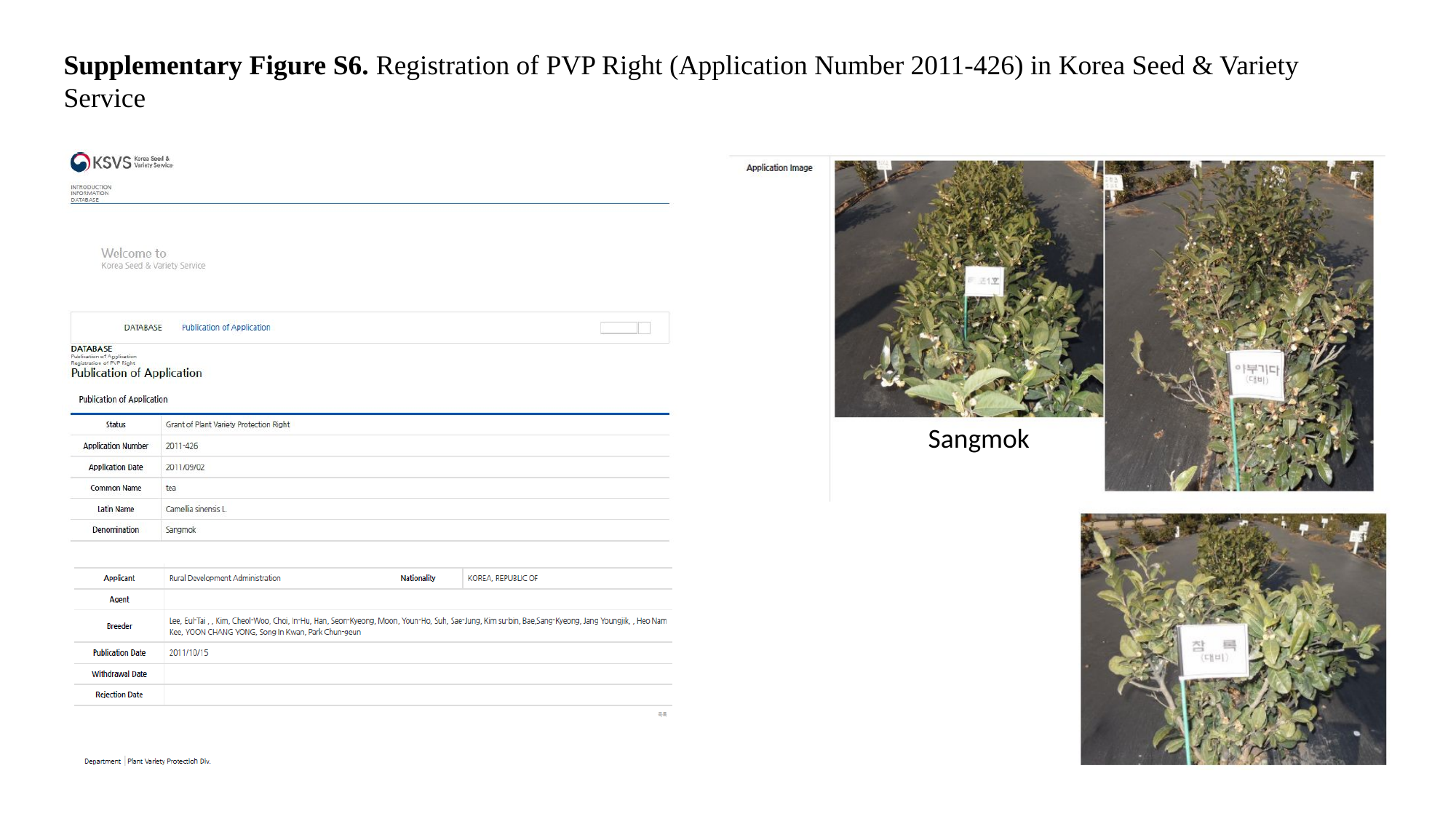

Supplementary Figure S6. Registration of PVP Right (Application Number 2011-426) in Korea Seed & Variety Service
Sangmok
